# Facial Expression Recognition Based on Fine-Tuned Channel–Spatial Attention Transformer

**DOI:** 10.3390/s23156799

**Published:** 2023-07-30

**Authors:** Huang Yao, Xiaomeng Yang, Di Chen, Zhao Wang, Yuan Tian

**Affiliations:** Faculty of Artificial Intelligence in Education, Central China Normal University, Wuhan 430079, China; yaohuang@ccnu.edu.cn (H.Y.); yxm_ccnu@mails.ccnu.edu.cn (X.Y.); chendi@ccnu.edu.cn (D.C.); wangzhao@mails.ccnu.edu.cn (Z.W.)

**Keywords:** facial expression recognition, attention, transformer

## Abstract

Facial expressions help individuals convey their emotions. In recent years, thanks to the development of computer vision technology, facial expression recognition (FER) has become a research hotspot and made remarkable progress. However, human faces in real-world environments are affected by various unfavorable factors, such as facial occlusion and head pose changes, which are seldom encountered in controlled laboratory settings. These factors often lead to a reduction in expression recognition accuracy. Inspired by the recent success of transformers in many computer vision tasks, we propose a model called the fine-tuned channel–spatial attention transformer (FT-CSAT) to improve the accuracy of recognition of FER in the wild. FT-CSAT consists of two crucial components: channel–spatial attention module and fine-tuning module. In the channel–spatial attention module, the feature map is input into the channel attention module and the spatial attention module sequentially. The final output feature map will effectively incorporate both channel information and spatial information. Consequently, the network becomes adept at focusing on relevant and meaningful features associated with facial expressions. To further improve the model’s performance while controlling the number of excessive parameters, we employ a fine-tuning method. Extensive experimental results demonstrate that our FT-CSAT outperforms the state-of-the-art methods on two benchmark datasets: RAF-DB and FERPlus. The achieved recognition accuracy is 88.61% and 89.26%, respectively. Furthermore, to evaluate the robustness of FT-CSAT in the case of facial occlusion and head pose changes, we take tests on Occlusion-RAF-DB and Pose-RAF-DB data sets, and the results also show that the superior recognition performance of the proposed method under such conditions.

## 1. Introduction

Facial expression is one of the most direct signals for expressing inner emotions in human communication. We can gain insights into a person’s physical or mental state by analyzing facial expressions. Therefore, facial expression recognition is of great significance in various fields, such as autonomous driving, human–computer interaction, and healthcare. It has gradually become an increasingly important research direction.

In human–computer interactions, machines utilize facial expression information to provide intelligent responses and enhance the overall interaction process. In driver fatigue monitoring, facial expression recognition is employed to detect the driver’s mental state and ensure safe vehicle operation, thereby issuing a timely reminder if necessary. In medical diagnosis, the facial expression recognition system analyzes the patient’s emotional state to provide additional support in treatment. However, automatic facial expression recognition still faces many challenges at present. Due to differences in gender, race, age, culture, and other factors, even for the same facial expression, there may be significant differences in the way emotions are expressed by different individuals. The features of facial expressions are interfered with by many other information, making facial expression recognition difficult. Additionally, in real life, there are often pose changes, occlusion, and other issues that will hamper the practical application of expression recognition. Thus, it is necessary to conduct further in-depth research on expression recognition to overcome these obstacles.

With the development of deep learning, convolutional neural networks (CNNs) have made remarkable achievements in the field of expression recognition. However, when the key facial parts are obstructed or there are pose changes, the performance of CNNs often decreases. CNNs extract features by continuously stacking convolutional layers, which can be computationally expensive and prone to the challenge of vanishing gradients, thus hindering network convergence. In recent years, transformer-based models, such as Vision Transformers (ViTs) [1] and Data-efficient image Transformers (DeiTs) [2], have achieved significant success in various computer vision tasks. Inspired by these achievements, Transformer has also been introduced into FER tasks [3,4].

ViT [1] is the first study to replace the CNN model with the transformer model and apply it to image classification. Inspired by ViT, Convolutional Visual Transformers (CVT) [5] is the first work that applies the transformer model to facial expression recognition. CVT uses the pre-trained ResNet18 as the backbone to extract feature maps from both the face image and LBP feature image. All the extracted features are then fused via attentional selective fusion (ASF). Subsequently, the spatial dimensions of the feature maps are flattened and projected to the specific dimension. Finally, the multi-layer transformer encoder is employed in classification. DeiT [2] introduces a new distillation procedure based on a distillation token and obtains competitive results. Mask Vision Transformer (MVT) [4] proposes a novel pure transformer-based model called MVT to solve FER tasks in the wild. The proposed mask generation network (MGN) can effectively filter out the backgrounds and interference of face images. Comprehensive experiments demonstrate the effectiveness and robustness of the proposed method. Dong et al. [6] proposed CSWin Transformer. It is an efficient and effective transformer-based backbone for general-purpose vision tasks. The self-attention mechanism in CSWin Transformer allows the model to capture dependencies between pixels across the entire image and enables the model to have a better understanding of the spatial relationships between different regions in an image.

Although the CSWin Transformer network can effectively extract local expression information, its self-attention mechanism divides the feature image into smaller steps for processing, which leads to limited learning ability for global feature information in the model. Furthermore, the models based on transformer have a larger number of parameters, longer training time, and greater difficulty in training and transfer compared to CNN-based models. Although CSWin Transformer is proven to achieve state-of-the-art performance across various vision tasks, it has a large number of parameters, demanding substantial computational resources and extensive training data and time. Additionally, CSWin Transformer typically performs well when trained on large datasets to capture a wide range of image variations. When applied to small datasets, it may suffer from overfitting or struggle to learn meaningful features. 

To address the dilemma between improving recognition accuracy and reducing computational complexity, this paper proposes an improved network model based on CSWin Transformer [6] for FER. Specifically, the CSWin backbone network is used to enhance local attention while introducing a channel–spatial attention module to provide better global attention. The fine-tuning method is then used to further improve the model’s recognition ability while reducing training parameters. The contributions of this article are as follows:

(1) We propose a fine-tuned channel–spatial attention transformer model (FT-CSAT) which integrates the channel–spatial attention module. This enhancement allows the model to not only focus on local information but also improve its capability to extract global features.
(2) To further improve the performance of FT-CSAT in the FER task, we insert a fine-tuning module after multi-layer perception (MLP), Cross-Shaped Window Self-Attention, and layer normalization (LN) operations. This approach improves both the performance of FT-CSAT and effectively controls the number of parameters.
(3) We evaluate the effectiveness and efficiency of our proposed FT-CSAT. Experimental results show that the recognition accuracy of FT-CSAT outperforms previous state-of-the-art methods on two commonly used datasets. (88.61% on RAF-DB and 89.26% on FERPlus.) Remarkably, the parameters of our model are only 27.35 M, which is smaller than other transformer-based models. In addition, even under the conditions of occlusion and pose changes, our model can still achieve good recognition accuracy.

The remainder of this paper is organized as follows: Section 2 reviews the related work on FER. In Section 3, we provide the details of the proposed method. Section 4 reports the experiment results on commonly used databases, followed by the conclusions in Section 5. 

## 2. Related Work

Since 2012, many classic CNN models have emerged, such as AlexNet [7], VGGNet [8], GoogLeNet [9], ResNet [10], etc. These models perform well in image recognition tasks. Therefore, researchers applied deep learning technology to facial expression recognition and achieved very good results. Luan Pham et al. [11], referring to the design of ResNet, designed an improved ResNet model. The model uses residual structure to solve the problem that the deeper the network depth may cause convergence problems. Mollahossein et al. [12] designed an expression recognition method based on the VGG model, which extracts expression features from the VGG model and achieves higher accuracy than previous methods. Jung et al. [13] proposed a recognition method for image expression sequences. The appearance features and geometric features are extracted from two different CNN networks, and the improved feature fusion method is used to fuse the two features. Yang et al. [14] designed a residual expression feature learning method, which uses the generative adversarial network (GAN) to convert different expression images into neutral expressions to learn expression features. The CNN model is used to classify the expression features learned by the GAN model. Lopes et al. [15] combined a simple convolutional neural network with specific image preprocessing techniques to improve the accuracy of expression recognition. Liu et al. [16] proposed an AU-inspired Deep Network (AUDN) inspired by facial motion units. AUDN decomposes a face into multiple facial action units and then uses CNN to extract features from these action units.

The application of CNNs has made significant progress in facial expression recognition. However, the convolutional filters in CNNs rely on local neighborhood operations, which lack global information and cannot capture long-distance dependencies between different facial regions. Facial expression recognition based on convolutional neural networks still has limitations. Therefore, Google researchers [17] proposed the transformer model in 2017, which makes up for the lack of global information in CNNs and achieves great success in machine translation and other tasks. ViT [1] achieves good classification results in image classification tasks by pre-training on large datasets and fine tuning on smaller datasets. The global self-attention mechanism of the Vision Transformer enables the network to overlook the impact of information-missing regions, guiding it to learn robust facial features from a global perspective. This enhances the network’s feature extraction capabilities, making it more powerful in capturing essential facial characteristics. Fuyan Ma et al. [5] were the first to propose the application of a visual transformer to expression recognition. They used an attention-selective fusion module (ASF) to aggregate both global and local facial information and guide the backbone network to extract the required information. Aouayeb et al. [18] proposed to combine the visual transformer with the SE attention module for expression recognition and achieved an accuracy of 87.22% on the RAF-DB dataset. Feng et al. [19] employed a greedy strategy to locally optimize the SWin transformer [20], fine-tuned the parameters, and their proposed model exhibits significant improvements over the baseline network in RAF-DB, FER2013PLUS, and AffectNet [21]. Dong et al. [6] proposed a Cross-Shaped Window self-attention mechanism and Locally enhanced Positional Encoding (LePE) to improve the performance on common vision tasks. The presented CSWin Transformer achieves state-of-the-art performance on various vision tasks under constrained computation complexity.

## 3. Method

We propose the fine-tuned channel–spatial attention transformer model (FT-CSAT), an efficient and effective transformer backbone consisting of two key modules: the channel–spatial attention module and the fine-tuning module. In this section, we first introduce the overall architecture of our model. Then, we describe these two modules in detail.

### 3.1. The Proposed Framework

**Figure 1:**
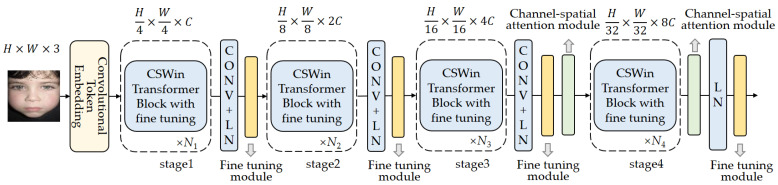
The proposed framework.

The overall architecture of the proposed framework is illustrated in Figure 1. CSWin Transformer is used as the baseline network. Although the CSWin Transformer network can effectively extract local facial expression information, its self-attention mechanism divides the input feature map into smaller step-sized blocks for processing, which hinders its ability to learn global feature information. To address this limitation, we introduce a channel spatial attention module to enhance the model’s extraction of crucial global information. To improve the training efficiency, the pre-training parameters of the CSWin Transformer on the ImageNet-1K data set are loaded, the cross-entropy function is selected as the loss function, and the Adam algorithm is used for backpropagation to optimize the model. ImageNet-1K is a subset of the large dataset ImageNet. It contains approximately 1.2 million images with 1000 categories. Categories in ImageNet-1K cover a wide range of objects and scenes, such as animals, vehicles, people, and natural landscapes. However, commonly used expression recognition datasets, such as RAF-DB and FERPlus, often consist of only six or seven types of expressions with tens of thousands of images. The data distribution of the expression data set is significantly different from that of the upstream data set ImagNet-1K. In order to further improve the performance of the model in downstream tasks, it is necessary to fine tune the pre-training parameters to better adapt to facial expression recognition in natural environments. Incorporating the pre-training parameters from ImageNet-1K allows the CSWin Transformer model to leverage the knowledge acquired from a large-scale dataset, enabling it to capture general image features and improve its performance on subsequent tasks. This approach often leads to faster convergence during fine tuning or training on smaller, task-specific datasets, as the model already possesses a strong initial understanding of visual patterns and concepts. Therefore, we adopt the parameter fine-tuning method to enhance the model’s performance while reducing the number of parameters.

### 3.2. Channel–Spatial Attention Module

In recent years, the attention mechanism, as one of the important components of neural networks, has been widely used in the field of expression recognition. Hu et al. proposed the SE (Squeeze Extraction) module [22], which learns the correlation relationships between various channels in the feature map, generates channel attention, and enables the network to focus more on informative channels. It brings significant performance improvements to CNNs. Convolutional Block Attention Module (CBAM), based on the attention mechanism of SE, was first proposed by Woo et al. [23]. Compared with SENet, which only focuses on channel features, CBAM is an attention module that combines channel attention and spatial attention. It adaptively adjusts features along two independent dimensions.

Suppose the input feature image is F and the dimension is C×H×W. The module will obtain channel attention map $M_{C}$ and spatial attention map $M_{S}$ via channel and spatial attention modules successively. The specific process can be summarized as follows:

(1) $$\left\{\begin{array}{c} F^{\prime }=M_{C}\left(F\right)⊗F\\ F^{\prime \prime }=M_{S}\left(F^{\prime }\right)⊗F^{\prime } \end{array}\right.$$

where ⊗ represents element by element multiplication, $F^{\prime }$ is the corrected output of the channel attention module, and $F^{\prime \prime }$ is the final output corrected in the spatial attention module.

The channel attention module is used to focus on whether there are target features in the input image. First, average pooling and maximum pooling are performed, respectively, to obtain $F_{avg}^{C}$ and $F_{max}^{C}$. Then, $MLP(F_{avg}^{C})$ and $MLP(F_{max}^{C})$ are obtained by weight sharing using the *MLP*. Finally, the feature map $M_{C}$ of channel attention is obtained using Sigmoid. The specific calculation formula is as follows:

(2) $$\begin{array}{c} M_{C}\left(F\right)=\sigma \left(MLP\left(Avgpool\left(F\right)\right)+MLP\left(Maxpool\left(F\right)\right)\right)\\ \;=\sigma \left(W_{1}\left(W_{0}(F_{avg}^{C})\right)+W_{1}\left(W_{0}(F_{max}^{C})\right)\right) \end{array}$$

where σ represents the Sigmoid activation function, and $W_{0}$ and $W_{1}$ represent the weight of *MLP*(·).

The spatial attention module mainly focuses on the feature information of the target’s location. Firstly, perform average pooling and maximum pooling operations on the feature $F^{\prime }$ generated by the channel attention module. Then, concatenate the two generated two-dimensional vectors and perform convolution operations. Finally, spatial attention feature $M_{S}\left(F^{\prime }\right)$ is generated using Sigmoid. The specific calculation formula is as follows:

(3) $$\begin{array}{c} M_{S}\left(F^{\prime }\right)=\sigma \left(f^{7\times 7}\left(\left[Avgpool\left(F^{\prime }\right),Maxpool\left(F^{\prime }\right)\right]\right)\right)\\ =\sigma \left(f^{7\times 7}([{F^{\prime }}_{avg}^{S};{F\prime }_{max}^{S}])\right) \end{array}$$

where *σ* represents the Sigmoid activation function, and f denotes the convolution operation with a kernel size of 7 × 7.

This paper proposes to add the attention module CBAM to the CSWin Transformer network so that the network can extract effective features from channel and spatial dimensions and suppress invalid features. It will guide the model to identify the key areas related to expression, thereby improving the feature learning ability of the model.

Discussion. CSWin Transformer network, used as the baseline in our paper, consists of four stages. Each stage consists of $N_{i}$ sequential CSWin Transformer Blocks and maintains the number of tokens. A convolution layer (3 × 3, stride 2) is used between two adjacent stages to reduce the number of tokens and double the channel dimension. CBAM is an end-to-end universal module that can be seamlessly integrated into any position of a convolutional neural network for end-to-end training. Theoretically, CBAM can be integrated into any stage of CSWin Transformer. The main difference lies in the size and size of the feature graph of each stage of the network. However, in fact, the integration of CBAM into different stages of CSWin Transformer will have different impacts on the accuracy of expression recognition.

**Figure 2:**
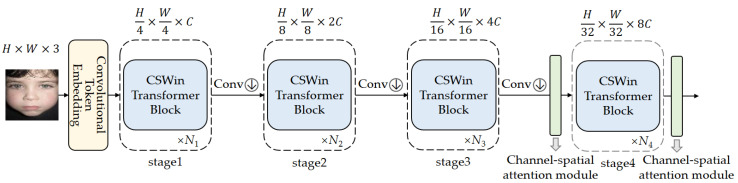
Network structure of the four experiments.

We conduct four integration approaches, as illustrated in Figure 2. Method (a) integrates CBAM into the first stage of the CSWin Transformer, method (b) into the second stage, method (c) into the third stage, and method (d) into the fourth stage. The recognition accuracy of these different methods on the RAF-DB and FERPlus expression datasets is presented in Table 1.

From Table 1, it can be observed that, except for method (a), the expression recognition accuracy of all other methods has improved. On the RAF-DB dataset, method (d) achieved the highest recognition accuracy, reaching 87.58%, which is 0.29% higher than the baseline. On the FERPlus dataset, method (c) achieved the highest recognition accuracy, reaching 88.05%, which is 0.38% higher than the baseline. Therefore, we propose method (e), which integrates the CBAM module into the third and fourth stages of the CSWin Transformer simultaneously. The experimental results demonstrate that method (e) achieves higher expression recognition accuracy. The accuracy on the RAF-DB and FERPlus datasets increased from 87.29% and 87.67% of the baseline to 88.01% and 88.51%, respectively.

Experiments show that after integrating the CBAM module into the CSWin Transformer network, the maximum pooling and average pooling in the channel domain and spatial domain of the CBAM module can effectively learn discriminative global and local features from facial expression images and accurately calculate the weight of each spatial position in the feature map, thus strengthening the role of important spatial features in the feature map in FER tasks.

### 3.3. Fine Tuning Module

The existing fine-tuning methods are mainly divided into two types. One approach is full fine tuning. This method tunes all parameters of the pre-training model, which inevitably leads to the introduction of more parameters. The other is to tune the last linear layer, which solves the problem of introducing too many parameters, but the accuracy is significantly reduced compared with full fine-tuning. This paper uses the Scaling and Shifting Features (SSF) parameter fine-tuning method [24]. Different from the above two methods, SSF can not only improve the performance of the model by fine tuning parameters but also controlling the number of parameters introduced.

The SSF parameter fine-tuning method can achieve parameter fine tuning only by scaling and shifting the deep features extracted using the pre-trained transformer model without introducing additional inference parameters. It draws on the concepts of variance and mean value. Pre-trained models trained on upstream datasets exhibit better feature extraction capabilities through scale and shift parameters. During the training of the downstream data set, the pre-training weight will be frozen, and the parameters will be updated until the feature input of SSF module. The feature output from the previous operation is performed dot product with a scale factor and then summed with a shift factor. The specific calculation formula is as follows:

(4) y=γ⊙x+β

where $x\in R^{(N^{2}+1)d}$ represents the input. $y\in R^{(N^{2}+1)d}$ is the output (is also the input of the next operation). $\gamma \in R^{d}$ and $\beta \in R^{d}$ are the scale and shift factors, respectively. ⊙ is the dot product.

**Figure 3:**
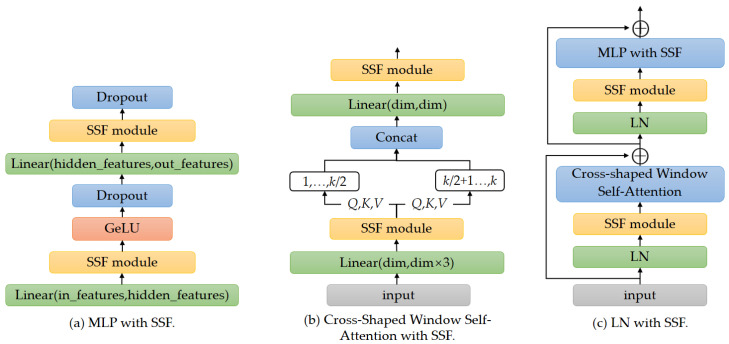
The specified operations with SSF.

In this paper, SSF is inserted after some specified operations in the pre-training model to modulate features, as shown in Figure 3. These specified operations include MLP, Cross-Shaped Window Self-Attention, and LN.

MLP with SSF. In the CSWin Transformer block, MLP consists of two fully connected layers, allowing the model to capture more complex relationships between image features and serve as the input for the next attention block. The output features of the fully connected layer are performed dots product with a scale factor and then summed with a shift factor. The MLP after inserting SSF is shown in Figure 3a. The specific calculation formula is as follows:

(5) $$y=\gamma ⊙\left(\omega *t+b\right)+\beta =\left(\gamma ⊙\omega \right)*t+\gamma ⊙b+\beta$$

where t is the input of the previous fully connected layer in the MLP, ω is the weight, and b is the bias. γ and β are the scale and shift factors, respectively.

Cross-Shaped Window Self-Attention with SSF. In CSWin Transformer, the Cross-Shaped Window Self-Attention mechanism based on the multi-head self-attention mechanism is proposed. The input is linearly transformed into a query *Q*, a key *K*, and a value *V*. In this paper, the output of the linear conversion is fine tuned using the SSF method. Then, the input features are linearly projected to *K* heads. The *K* heads are equally split into two parallel groups (each has *K*/2 heads). The 1, …, *k*/2 heads perform horizontal stripes self-attention. The *k*/2 + 1, …, *k* heads perform vertical stripe self-attention. The output of these two parallel groups will be concatenated back together by a fully connected layer. In this paper, the SSF modules are inserted after the fully connected layers. The Cross-Shaped Window Self-Attention with SSF is shown in Figure 3b.

LN with SSF. LN is used to normalize the output features of the CSWin-Attention, which helps to stabilize the training process and improve the performance of the model. Each CSWin Transformer block contains two LNs. As shown in Figure 3c, we insert SSF module after each LN operation for parameter fine tuning. During the fine-tuning process, the pre-trained LN weight parameters are frozen, the scale factor and shift factor are updated and then merged into the original parameter space.

## 4. Experimental Results

### 4.1. Implementation Details

To evaluate the performance of our method for expression recognition in natural scenes, experiments are conducted on the RAF-DB dataset [25] and FERPlus dataset [26]. In the experiments, we use the training and validation sets together as the training network and add random Gaussian noise to the images for data enhancement, keeping the test set unchanged. During the experiment, we used the maximum voting method, where the expression category of each image with the highest number of expression labels was the expression category label of the image. In order to verify that our method can effectively obtain facial expression information when posture changes, facial detection, cropping, and alignment processing are not performed on these two datasets in the experiment.

In this paper, we use CSWin Transformer-T (Tiny) as the baseline network, and the pre-trained parameters on the ImageNet-1K dataset are loaded. The derivatives of each parameter are calculated using the cross-entropy loss function, and the parameters are updated with the Adam optimization algorithm. The initial learning rate is 0.00009, and the batch size is 32. We train our model on an NVIDIA GeForce GTX 3080Ti GPU with 12 GB RAM.

### 4.2. Comparison with State-of-the-Art Methods

Evaluation of RAF-DB: At present, there are two popular methods to solve the facial expression recognition problem: one is based on CNN, and the other is based on a Vision Transformer. We compare our method with CNN-based methods, including RAN [27], SPWFA-SE [28], OADN [29], SCN [30], IF-GAN [31], and EfficientFace [32]. We also compare our method with the recently proposed methods that use a hybrid model of the transformer and the CNN, such as CVT [5], PACVT [33], and FER-VT [34]. Table 2 reports the results of our method compared with previous methods on the RAF-DB dataset. Our method yields the highest score of 88.61% in accuracy, which is 0.25% better than the second-best method based on CNNs and 0.35% better than the second-best hybrid method separately.

Evaluation of FERPlus: Table 3 evaluates our method with previous methods on the FERPlus dataset. We compare our method with recent state-of-the-art methods, including LDR [35], RAN [27], SCN [30], CVT [5], MVT [4], and Meta-Face2Exp [36]. As we can see in Table 3, our method achieves the best accuracy of 89.26% on the FERPlus dataset, which is 0.38% higher than the second-best method (MVT [4]).

Evaluation of parameters: To demonstrate that our method only needs to introduce a small number of adjustable parameters, Table 4 compares the total parameter number of our model with the existing facial expression recognition methods, including CNN-based models and transformer-based models.

As can be seen, our parameter number is 27.346 M less than the 51.8 M of CVT [5], which also uses a transformer backbone network. The ELSA-SWin-T [40] model replaces W-MSA and SW-MSA in SWin Transformer Block with ELSA, which improves the performance of local attention. The number of parameters of ELSA-SWin-T is comparable to that of SWin-T, but our model still has fewer parameters than either of these models.

Evaluation of occlusion and variant pose: To further validate the effectiveness of our proposed method in addressing occlusion and head pose changes in natural scenes, we conduct experiments on the Occlusion-RAF-DB and Pose-RAF-DB datasets.

As shown in Table 5, although our method is not specifically designed for occlusion and variant pose FER issues, our method outperforms RAN [27] and CVT [5] in each case, which shows the superiority of our method. Specifically, our method exceeds RAN and CVT by 2.41% and 1.18% on Occlusion-RAF-DB, respectively. Our method also outperforms RAN and CVT on Pose-RAF-DB. It exceeds RAN by 1.25% and 3.23% with poses larger than 30 degrees and 45 degrees, respectively. It exceeds CVT by 0.02% and 0.17% with poses larger than 30 degrees and 45 degrees, respectively. In summary, our method proposed in this paper outperforms the previous methods for face occlusion and head pose variation in natural scenes. It can effectively enhance the robustness of the model to both occlusions and pose variation problems.

### 4.3. Visual Analysis

**Figure 4:**
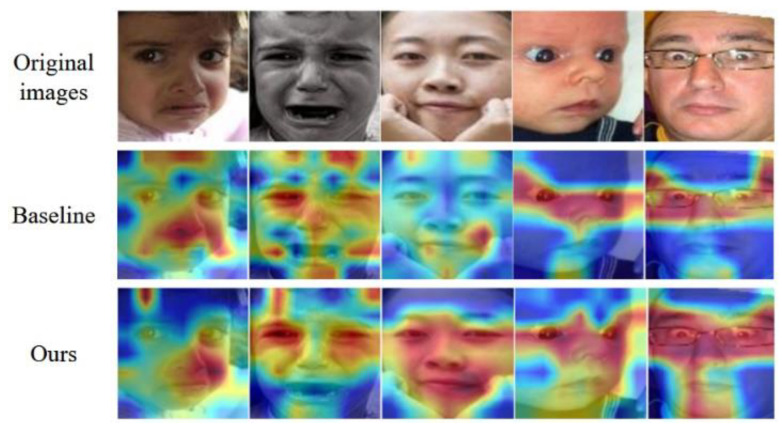
Attention visualization.

To further investigate the effectiveness of our approach, we employ the Grad-CAM method [41] to visualize the attention maps generated by our model. Figure 4 shows the attention maps of different emotions in RAF-DB. The baseline is CSWin Transformer-Tiny pre-trained on ImageNet-1K. The first row shows the original facial images, and the second to third rows show the results of the baseline and our method, respectively.

The darker colors indicate high attention, while the lighter colors indicate less attention from the model. From the resulting images, it can be seen that the model proposed in this paper can better perceive global attention information.

### 4.4. Ablation Study

In order to verify the validity of each module in the proposed model, ablation experiments are conducted on RAF-DB and FERPlus datasets, as shown in Table 6. Specifically, the model takes the CSWin-T pre-trained on the ImageNet-1K data set as the baseline network for the experiment and then adds the channel–spatial attention module and the fine-tuning module to the baseline network, respectively. In the experiment, the same training settings are used for training. When global feature information is extracted using the channel-space attention module, the recognition accuracies on RAF-DB and FERPlus are increased by 0.72% and 0.84%, respectively, compared with the baseline network. In addition, although the number of parameters increased by 5 MB after fine tuning, the accuracies on RAF-DB and FERPlus further improved by 0.6% and 0.75%, respectively.

## 5. Conclusions

In this paper, we introduce FT-CSAT, a model based on CSWin Transformer for the facial expression recognition (FER) task. Our approach involves the incorporation of a channel–spatial attention module to enhance the model’s global feature extraction capabilities. Additionally, we employ a fine-tuning method to further optimize the model’s performance and control the number of introduced parameters. Experimental results demonstrate the superiority of FT-CSAT over other state-of-the-art methods on two widely used facial expression datasets, namely RAF-DB and FERPlus. Furthermore, experiments conducted on the Occlusion-RAF-DB and Pose-RAF-DB datasets showcase the robustness of our method in handling facial occlusion and head pose changes.

In future work, the proposed method can be further improved in the following respects: (1) To further address the issue of facial expression recognition caused by changes in facial scale, the multi-resolution strategy [42] can be considered to extract reliable and stable facial features. (2) It is necessary to establish a high-quality and large-scale facial expression database to reduce the problems of uneven distribution of expression images and inconsistent expression category labeling as much as possible to improve the performance of advanced deep-learning models in FER tasks.

## Figures and Tables

**Table 1:** Different experiments.

Methods	Acc. (%)	
	RAF-DB	FERPlus
baseline	87.29	87.67
(a) stage 1	87.19	87.65
(b) stage 2	87.42	87.86
(c) stage 3	87.52	88.05
(d) stage 4	87.58	87.95
(e) stage 3 and stage 4	88.01	88.51

**Table 2:** Comparison on RAF-DB.

Type	Methods	Acc.
CNN	RAN [27]	86.90
	SPWFA-SE [28]	86.31
	OADN [29]	87.16
	SCN [30]	87.03
	IF-GAN [31]	88.33
	EfficientFace [32]	88.36
Transformer + CNN	CVT [5]	88.14
	PACVT [33]	88.21
	FER-VT [34]	88.26
Transformer	The proposed method	88.61

**Table 3:** Comparison on FERPlus.

Methods	Year	Acc.
LDR [35]	ICIP 2020	87.60
RAN [27]	TIP 2020	87.85
SCN [30]	CVPR 2020	88.01
CVT [5]	IEEE Trans 2021	88.81
MVT [4]	2021	89.22
Meta-Face2Exp [36]	CVPR 2022	88.54
The proposed method	-	89.26

**Table 4:** Comparison of parameters.

Type	Methods	Params
CNN	gACNN [37]	>134.29 M
	TAMNet [38]	51.67 M
Vision Transformer	CVT [5]	51.80 M
	D-DW-Conv.-T [39]	51 M
	SWin-T [20]	28.3 M
	ELSA-SWin-T [40]	29 M
	The proposed method	27.35 M

**Table 5:** Results on Occlusion-RAF-DB and Pose-RAF-DB.

Methods	Occlusion	Pose (30)	Pose (45)
RAN [27]	82.72	86.74	85.2
CVT [5]	83.95	87.97	88.26
The proposed method	85.13	87.99	88.43

**Table 6:** Ablation experiment results on RAF-DB and FERPlus. In the table, × indicates that the module is not included in the model, and √ indicates that the module is included in the model.

Channel–Spatial Attention Module	Fine-Tuning Module	Acc. (%)		Params
		RAF-DB	FERPlus	
×	×	87.29	87.67	22.32 M
√	×	88.01	88.51	22.35 M
√	√	88.61	89.26	27.35 M

## Data Availability

Data underlying the results presented in this paper are available in RAF-DB [25] and FERPlus [26].
